# Photoinduced modulation and relaxation characteristics in LaAlO_3_/SrTiO_3_ heterointerface

**DOI:** 10.1038/srep08778

**Published:** 2015-03-05

**Authors:** K. X. Jin, W. Lin, B. C. Luo, T. Wu

**Affiliations:** 1 Shaanxi Key Laboratory of Condensed Matter Structures and Properties, School of Science, Northwestern Polytechnical University, Xi'an 710072, China; 2 Materials Sciences and Engineering, King Abdullah University of Science and Technology, Thuwal 23955-6900, Saudi Arabia

## Abstract

We report the modulation and relaxation characteristics in the two-dimensional electron gas system at LaAlO_3_/SrTiO_3_ heterointerface induced by the ultraviolet light illumination (365 nm). The suppression of Kondo effect at the interface illuminated by the light originates from the light irradiation-induced decoherence effect of localized states. It is interesting to note that the persistent and transient photoinduced effects are simultaneously observed and the photoinduced maximum change values in resistance are 80.8% and 51.4% at *T* = 20 K, respectively. Moreover, the photoinduced relaxation processes after the irradiation are systematically analyzed using the double exponential model. These results provide the deeper understanding of the photoinduced effect and the experimental evidence of tunable Kondo effect in oxides-based two-dimensional electron gas systems.

The transition metal oxide heterostructures have attracted considerable attentions in view of finding novel devices in the emergent arena of oxide electronics and microelectronics beyond the conventional semiconductor technology^1^,^2^. In these epitaxial oxide heterostructures, the (001) heterointerface between LaAlO_3_ (LAO) and SrTiO_3_ (STO)^3^,^4^,^5^ is the most fascinating one. A high-mobility two-dimensional electron gas (2*DEG*) with the order of 3 × 10^14^ cm^−2^ is observed at the interface although both materials in bulk form are conventional insulators^6^,^7^,^8^. Subsequently, presences of the 2*DEG* at LaGaO_3_/STO^9^, DyScO_3_/STO^10^, NdAlO_3_/STO^11^, GdTiO_3_/STO^12^ and NdGaO_3_/STO interfaces^13^, have been experimentally revealed despite the rare 2*DEG* systems in oxide heterointerfaces. Until now, the heterointerfaces exhibit a wide range of intriguing physical phenomena and properties, such as superconductivity^14^,^15^, large negative magnetoresistance^16^, ordered magnetic ground states^17^,^18^, the coexistence of ferromagnetism and superconductivity^19^,^20^,^21^, Kondo effect^22^,^23^, resistance switching^24^, and so on. Furthermore, the two-dimensional electron gas has the tunability of the conduction state by an atomic force tip^25^, electrostriction^26^, charge and orbital order^27^, and photovolatges^28^, *etc.* Meanwhile, the photoinduced effect, being the external perturbation of the heterointerface, provides a handy and reliable method to induce the change of properties or the insulator-metal transition^29^. Additionally, photoinduced effects are generally used to photogenerate a hidden electronic phase or the state of matter and unveil the intrinsic physical mechanisms^30^. Some photoinduced phenomena have been discovered in oxides with multiphase coexistence^31^,^32^,^33^. Moreover, the persistent and reversible phases have been observed in the charge-ordering thin film, promising for photonic device applications^34^. Actually, the photoinduced characteristics have been observed in some oxide heterointerfaces with 2*DEG* systems. For examples, Gennaro *et al.* and Tebano *et al.* have studied the persistent photoconductivity at oxide interfaces^35^,^36^ and Chan *et al.* have enhanced photoresponse by using Pd nanoparticles^37^. Yamada *et al.* have deeply analyzed the localization dynamics in the photocarrier recombination process and discussed the interface effects on the electron relaxation dynamics in terms of the strong interface potential^38^. Irvin *et al.* have investigated the photoresponse on the nanoscale and developed the rewritable oxide photodetectors^39^. Lu *et al.* have found that the insulating state of the interface can be converted to metallic state by the light illumination and made the LAO/STO interface as a promising nonvolatile memory^40^. Rastogi *et al.* have investigated the perturbation on photoconducting state by electro static fields^41^. Here, we report the photoinduced phase transition originating from the suppression of Kondo effect and the relaxation process at the LAO/STO heterointerface with a 10 u. c. LAO layer prepared by pulsed laser deposition method^42^,^43^. Moreover, the persistent and transient photoinduced effects have been systematically investigated. Our results could be helpful for further understandings of the photoinduced phenomena in oxide heterostructures and potential applications in the all oxide electronics.

## Results and Discussion

**Figure 1:**
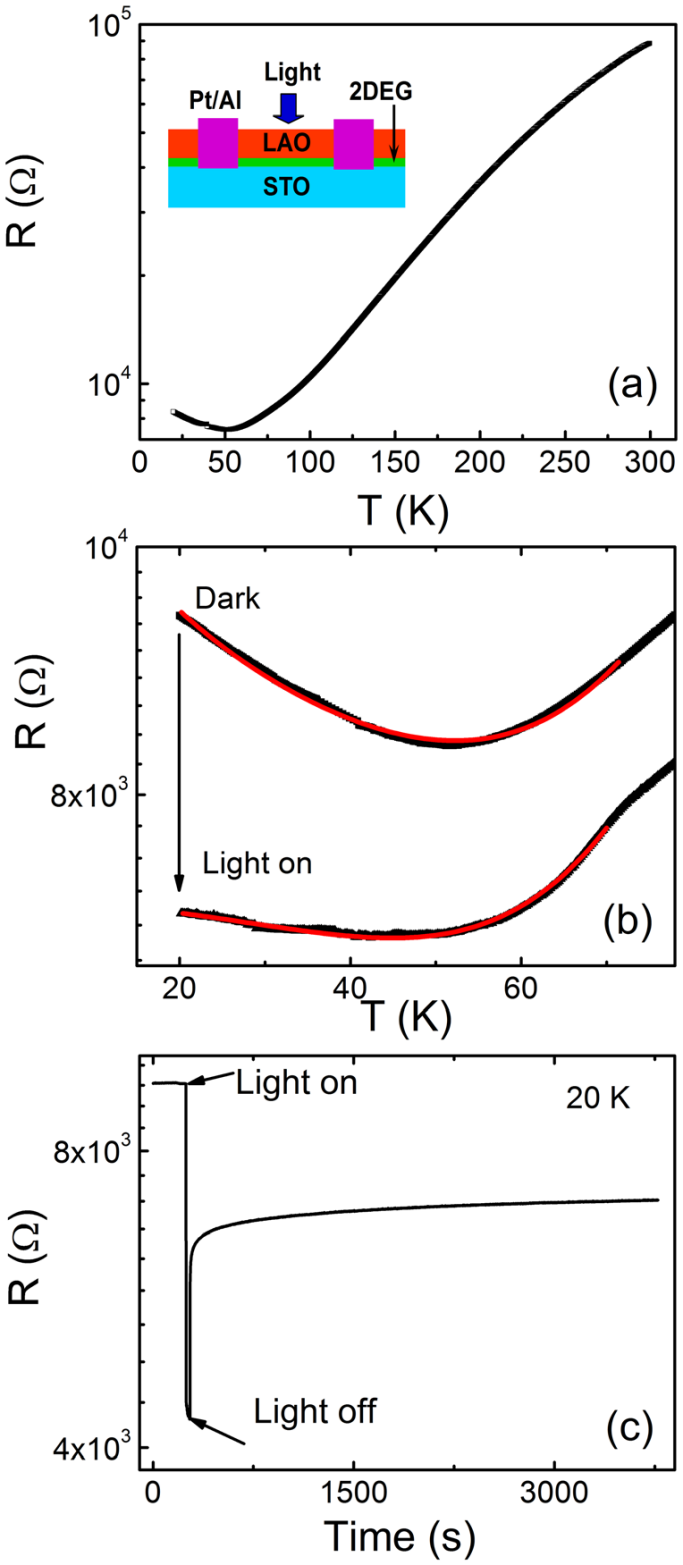
(a) Resistance-temperature curves of the heterointerface in darkness, and the inset displays the schematic illustration of the heterointerface for the photoinduced measurement. (b) Resistance versus temperature curves of the heterointerface with and without the light irradiation at *T* < 80 K. The solid lines are the fitted curves. (c) Time dependence of the resistance in the heterointerface at 20 K.

Figure 1 (a) shows the resistance-temperature curves of the heterointerface in darkness. The inset displays the schematic illustration of the heterointerface for the photoinduced measurements. It is observed that the heterointerface exhibits a typical metallic conduction at *T >* 55 K. When the temperature is lower than 55 K, the resistances are increased as the temperature is decreased. Namely, the sample displays an upturn phenomenon in the resistance of metallic systems at low temperatures, which can be regularly observed in some doped oxide semiconductors^44^ and the 2*DEG* systems^22^,^23^. This could be attributed to a Kondo effect^45^, one of the most possible effects in this system, indicating the obvious role of the magnetic interactions among localized and delocalized electrons at low temperatures. Here, the effect might arise from the interplay between the itinerant conduction electrons and localization electrons, which is an admixture composed of localized and unpaired electrons (likely polaronic in nature)^46^. The itinerant conduction electrons are localized at *T* < 55 K and as a result the concentration is deceased. The resistance versus temperature curves of the heterointerface in darkness and after the light irradiation at *T* < 80 K are presented in Figure 1 (b). It is interesting that the upturn phenomenon in the resistance basically disappears and the curve shows a slightly upward trend after irradiating the heterointerface by the light. The expression of the resistance-temperature curves of the heterointerface in darkness can be described by the expression^47^

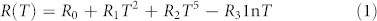
where *R*_0_ is the residual resistance due to the disorder, the second and the third terms represent the functional temperature dependence of the contributions from electron-electron and electron-photon interactions, respectively. According to Kondo theory, ln*T* is the contribution from the exchange coupling between the conducting electrons and the localization electrons. As the solid line in Figure 1(b) shows, the fitting curve agrees with the experimental data well. The obtained fitting parameters are *R*_0_ = 13487 Ω, *R*_1_ = 13.3*10^−3^ Ω/K^2^, *R*_2_ = 7.3*10^−7^ Ω/K^5^, *R*_3_ = 1348.9 Ω/lnK. After irradiating the sample, the Kondo effect is shrunk. Thereby the total expression of the resistance-temperature relation can be modified as 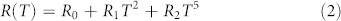
As shown in Figure 1 (b), the numerical fitting using Eq. (2) to the resistance-temperature curve yields *R*_0_ = 7265 Ω, *R*_1_ = 0.19 Ω/K^2^, and *R*_2_ = 8.7*10^−6^ Ω/K^5^. The Kondo effect at low temperature is suppressed by the photocarriers injection, similar to effects of the dopant and magnetic field^48^,^49^. The suppression effect might originate from the light irradiation-induced decoherence effect of localized states. At the LAO/STO interface, the orbital reconstruction due to the broken symmetry is well established. Particularly, the degeneracy of the Ti *t_2g_* state is lifted at the interface, and the Ti 3d_xy_ state possesses the lowest energy. Accordingly, the 3d_xy_-derived band is occupied firstly and the electrons are highly susceptible to the localization. When the light irradiates the heterointerface, the photocarriers induce the delocalization of electrons. Thus, the Kondo effect is suppressed and the photoinduced phase transition from the insulating to metallic state emerges at low temperatures. Similar results were found in a quantum dot induced by the external irradiation^50^.

As shown in Figure 1 (c), the resistances of the heterointerface are decreased from the 8.7 kΩ to 4.3 kΩ at *T* = 20 K when it is irradiated by the light. It is clear that the resistance is restored to a steady value and then maintains this value for the long time when the light is off. Namely, the heterointerface favors the persistent photoinduced effect. The conduction mechanisms of the 2*DEG* system have three possible explanations: the electronic reconstruction caused by the polar catastrophe, the La interdiffusion through the interface, and the defect generation (such as oxygen vacancies) in the STO substrate^51^. Here, we account for the photoinduced effect based on the electronic reconstruction scenario, which is a displacement of electrons from the outer region of LAO into the Ti 3d states of the topmost STO layers. And this is driven by the relaxation of the electrostatic energy accumulated in the polar layer, forming the built-in field. Thus, the polar layer plays an important role in the persistent photoinduced effect. Besides, the oxygen vacancies generated during the preparation of the film are inevitable and thus affect the photoindueced effect by the formation of the subbands. Likewise, the persistent photoconductivity is also observed in some semiconductor-based interfaces with a 2*DEG* system, which is explained in terms of a separation of the electron-hole pairs by the built-electric field^52^,^53^,^54^. The separation results in the long lifetime of recombination process. The photon energy (~3.3 eV) of the light with the wavelength of 365 nm is smaller than the band gap of LAO (~5.6 eV) and larger than that of STO (~3.2 eV)^55^. Thereby the light can irradiate the 2*DEG* interface at STO side and generate the electron-hole pairs by passing through the LAO layer, resulting in an increase in the 2*DEG* density. The excited electrons falling in the triangular interfacial potential well in STO side are added to 2*DEG* systems and holes are localized by shallow defects. When the light is off, the trapped electrons in the potential well will not recombine with holes due to the energy barrier. Thereby the persistent photoinduced effect occurs. The decay dynamics of photoinduced effects are determined by the lifetime of the recombination probability of the photoinduced electron-hole pairs. Actually, the STO single crystals also exhibit the persistent photoconductivity when exposed to subband gap light, which is attributed to the excitation of an electron from a titanium vacancy defect into the conduction band^56^. In this paper, the photon energy with the value of about 3.3 eV is slightly higher than the band gap of STO. Moreover, we have only found the transient photoinduced effect and an insulator-metal phase transition in a bare STO single crystal at the same condition^31^. This further confirms the effect of polar discontinuity at the interface.

**Figure 2:**
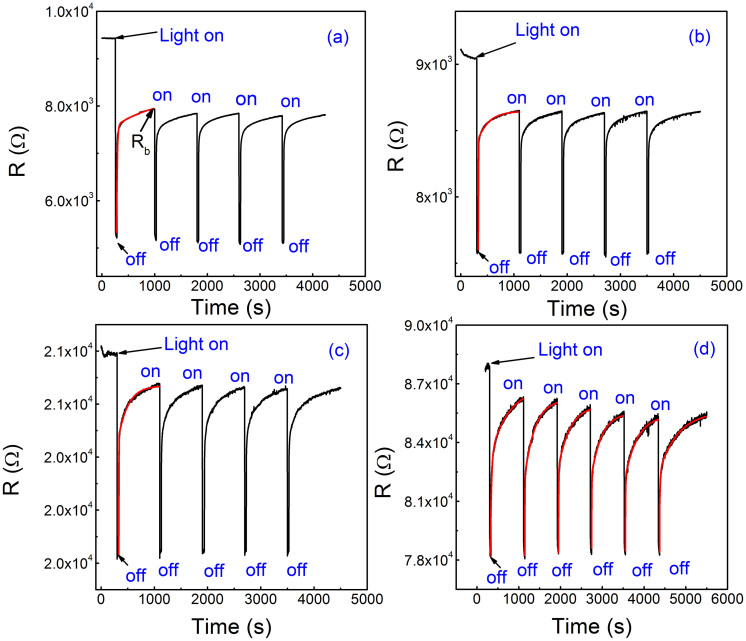
Time dependence of the resistance at different temperatures, (a) 20 K, (b) 80 K, (c) 160 K, (d) 300 K. The red solid lines are the fitting curves.

**Figure 3:**
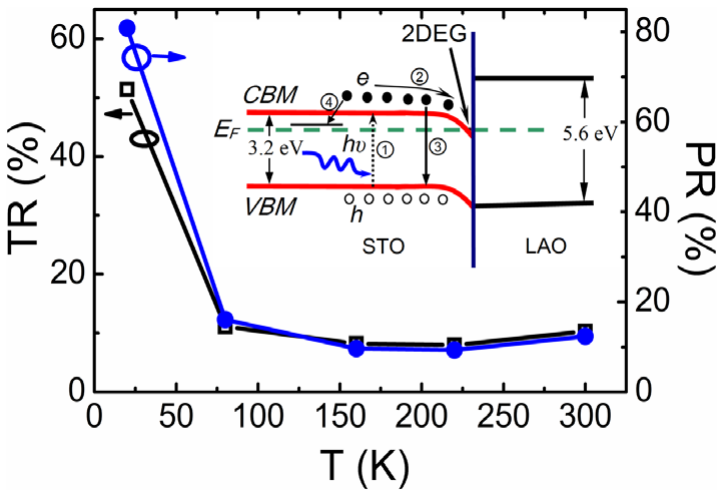
Persistent and transient photoinduced change in the resistance of heterointerface as a function of temperature. Inset: schematic diagram of band structure at the polar/non-polar interface where the band bending is displayed.

In order to further investigate the photoinduced response process, we performed the experiments on the time dependence of resistances. As shown in Figure 2, the time dependence of the resistance at different temperatures, (a) 20 K, (b) 80 K, (c) 160 K, and (d) 300 K is displayed. The resistances quickly decrease to the minimum values when the light is on, and then show a decay to a steady value when the light is off. It is worthwhile to note that the heterointerface under the first irradiation shows the persistent photoinduced effect and then restores to a balanced state. After that, the heterointerface exhibits the transient photoinduced effect and has the excellent repetition when the heterointerface is irradiated again. Here, the persistent photoinduced change in the resistance (*PR*) is defined as (*R*_0_-*R*_p_)/*R*_p_, where *R*_p_ is the resistance of the sample irradiated by the light, and *R*_0_ is the original resistance without the light irradiation. Meanwhile, the transient photoindcued change in the resistance (*TR*) is defined as (*R*_b_-*R*_p_)/*R*_p_, where *R*_b_ is the resistance of the balanced state after the first irradiation. The *PR* and *TR* dependences of temperature are shown in Figure 3. As we can see, the *PR* and *TR* effects strongly depend on the temperature. Both the values of the *PR* and *TR* show the same tendency and decease with increasing the temperature, which is ascribed to the thermal fluctuations at higher temperatures^57^. The maximum values of the *PR* and the *TR* are 80.8% and 51.4% at *T* = 20 K, respectively. Considering both the band bending in the polar layer as mentioned by the electronic reconstruction scenario^58^,^59^ and subbands in STO, the band diagram is displayed in the inset of Figure 3, providing a sketch of states at the interfaces. We can see that direct promotion from the valence band maximum (*VBM*) to the conduction band minimum (*CBM*) is made under the irradiation of 365 nm light (process 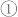). Then the photoexcited electrons drift to the channel region (process 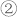), increasing the 2*DEG* density and then contributing to the observed decrease in the resistance. When the light illumination is off, the photoexcited electrons will be recombinated with holes at the *VBM* in STO (process 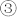) and be recaptured slowly by the subbands (process 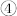) in the figure. Taking account into results above, we speculate that the photoinduced effect might contain two parts. One is the process that is not recovered, originating from the effect of polar layer at the 2*DEG* interface (process 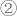). The other is the process, which can be recovered and related with the intrinsic STO. This includes the recombination of electrons in the conduction band and holes in the valence band of STO (process 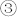) and recapture of electrons into the subbands (process 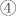) after the irradiation.

**Figure 4:**
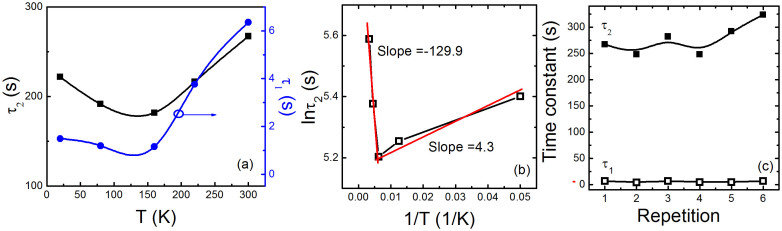
(a) Time constants of the heterointerface when the light is off as a function of temperature. (b) Logarithm of the time constant (*τ*_2_) as a function of reciprocal temperature. The red solid lines are the fitting curves. (c) Time constants dependence of repetition times.

To obtain insight into the persistent and transient photoinduced effect, we further analyzed the relaxation characteristics of the process when the light is off. The resistances increase nonlinearly with the irradiation time when the light is off at *T = * 20 K, 80 K, 160 K and 300 K as shown in Figure 2. The recovery of the resistance after the irradiation is the relaxation process of carriers, indicating the carrier's dynamics of recombination. The single exponential function can't describe the decay process very well and thus we make a fit using the double exponential function due to the two fitting parameters, which dominate the fast and slow processes, respectively. As shown in Figure 2, the resistance *vs.* time curves of STO after the irradiation can be fitted by the following formula:
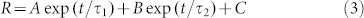
where *A* and *B* are the magnitude, *t* is the time, *τ*_1_ and *τ*_2_ is the fast and slow time constant of relaxation process, respectively. The time impendent term *C* is presumably due to the heating effect lasting for longer time than time range concerned here. Usually the band-band excitation is very fast and related with the initial excitation of electrons to higher energy states. A longer generation lifetime is mostly caused by a more complex process, involving the trapping and thermal activation processes. Hence, that exhibits probability for a lattice relaxation at the surface, in which shallow energy levels in the band gap form when shallow donors convert into deep donors. Such separation prevents the recombination process and leads to very long lifetimes of the photoexcited recovery. The exponential model provides an improved description of the photoinduced decay process. The red lines represent the fitting curves and a good agreement between the fitted and experimental data is obtained as shown in Figure 2. We can get the values of the time constant at different temperatures from the fitting. Figure 4 (a) shows the time constants of the heterointerface when the light is off as a function of temperature. It is observed that both the fast and slow time constants decrease to a minimum value at about 160 K firstly, and then are followed by an increase. The time constants are the characteristics of relaxation process in the photoinduced resistance change, and there exists the similar phenomena in the magnetization and the spin glasses of the manganites^60^,^61^. It is important to note that the temperature corresponding to the minimum value is roughly close to that of the cubic-to-tetragonal structural phase transition of STO (~105 K). So, we believe that such a phase transition is significant due to its latent paraelectric nature, which is intimately associated with the *d*^0^-ness of the Ti ions in STO because the light can irradiate the STO through the LAO layer. Additionally, the occupation of Ti with extra electrons being doped at interface is modified due to the polar layers. Therefore the interface also plays an important role although we expect the same to happen deep down in the substrate^62^. Meanwhile, the time decay is dependent on temperature. This behavior can be explained as the probability of the thermal activation of the localized carriers to overcome the potential barrier. Then the thermal carriers escape to the recombination channels. Thus, we express the temperature dependence of the slow time constant by following the Arrhenius-law^63^, which is expressed by: *τ*_2_ *= τ_0_e^−E/kT^*, where *τ_0_* is the high temperature limit of time constant, *E* is the thermally activated energy for the thermal capture of an electron at deep levels, and *k* is Boltzmann constant. Figure 4 (b) shows Arrhenius plot of *τ*_2_ with temperature. The red solid lines are the linear fitting curves and the time constants agree with the formula. Clearly, it is observed that two distinct temperature regions of activation process exist in the Arrhenius plot at about 160 K, which is consistent with the temperature dependence of the time constant. From the fitting, the calculated value of *E* is about 0.04 meV at low temperatures *T* < 160 K. However, at higher temperatures, the *E* is estimated as 1.12 meV, which is almost three times higher in comparison to the activation energy at low temperatures. The obtained values are approximately consistent with the previously reported values in oxides and GaN based 2*DEG* systems^62^,^64^. Nevertheless, the obtained values are smaller than that. The two-dimensional electron gas is thought to be formed from the three 3*d*-*t*_2*g*_ states. The energy scale for the electric subband states is expected to be of the order of tenths of an eV, determined by an interface electric field, electron density, and strain^65^. The energy scale in our experiments is on the scale of meV, which is attributed to the different depths of the localization at subbands of STO. The smaller activation energy means that the depth of localizations is shallower for our heterointerface. Considering the practical applications, we further analyze the characteristics of repetition at 300 K. Figure 4 (c) shows the time constants obtained from the fitting done using Figure 2 (d) dependence of repetition times. Both the fast and slow time constants slightly increase with increasing the repetition times, indicating the transient photoinduced effect has nice repetitions. We expect that the investigation following the results can enhance our understanding of the electrical and optical properties in 2*DEG* systems. In summary, we investigate the photoinduced modulation and relaxation characteristics in the two-dimensional electron gas system at LAO/STO heterointerface irradiated by the ultraviolet light (365 nm). The Kondo effect at the interface is suppressed by the light, which originates from the light irradiation-induced decoherence effect of localized states. The persistent and transient photoinduced effects are simultaneously observed and the minimum values of time constants gained from the double exponential model appear. These results would open the way for studying very fundamental solid state phenomena confined to an interface and suggest potential applications in the development of optical detectors using oxides-based devices.

## Methods

The 10 u. c. LAO film was grown on TiO_2_-terminated STO substrates at 800°C under an oxygen pressure of 10^−3^ mbar using the pulsed laser deposition method. The repetition rate of the KrF laser is 1 Hz and the fluence is 1.5 J/cm^2^. The film growth was monitored by in situ high-pressure reflection high-energy electron diffraction (RHEED). The RHEED intensity oscillation indicates a layer-by-layer growth mode. In order to obtain an Ohmic contact, the Pt/Al square-shaped electrodes with the size of 0.5 mm*0.5 mm were used as electrodes. The distance between two electrodes is about 0.5 mm. The sample was placed in a Janis CCS-300 closed-circuit refrigerator cryostat with quartz glass windows. The Keithley multimeter (model 2635A) was used for the resistance-temperature measurements in the temperature range from 20 to 300 K. The ultraviolet light with the wavelength of 365 nm and the power density of 2.6 W/cm^2^ typically illuminated the sample. Before the light illumination, the samples were kept in darkness for 10 h for the data measured until the resistance was stabilized.

## Author Contributions

K.X. and T.W. conceived and designed the experiments. W.L. prepared the sample. K.X. And B.C. performed the experiments. All authors discussed the results and conmented on the manuscript.
